# Supplementary material for: DMRforPairs: identifying Differentially Methylated Regions between unique samples using array based methylation profiles
Source: BMC Bioinformatics. 2014 May 15;15:141. doi: 10.1186/1471-2105-15-141 (PMC4046028; doi:10.1186/1471-2105-15-141)
Supplement: Additional file 2 — DMRforPairs output for the comparison of A431-MCF7 and NA17018-NA17105. Please start from the HTML files in each folder. Available via the BMC Bioinformatics website. [file 1471-2105-15-141-S2.zip › 1394847754114233_MOESM2_ESM/A431_MCF7/figures/12620.pdf]

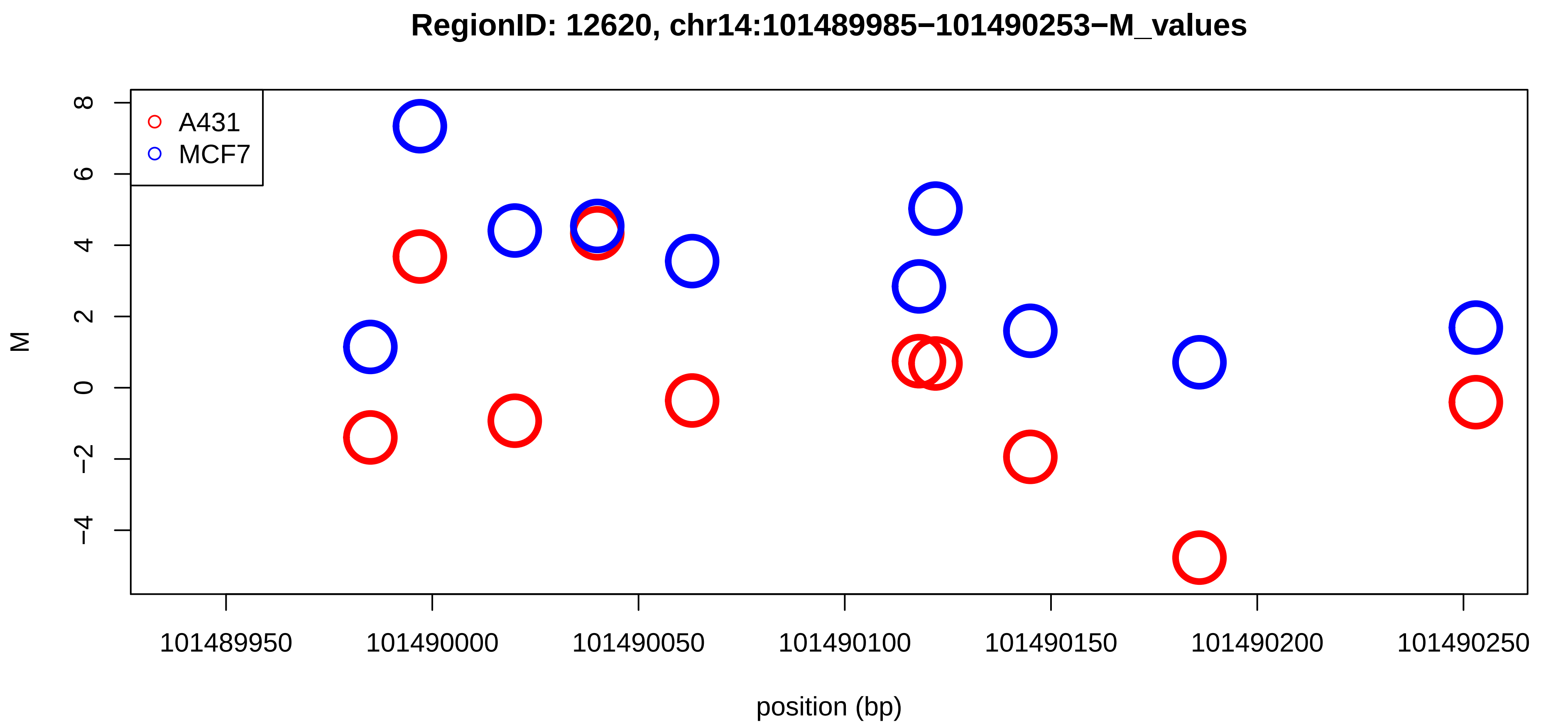
RegionID: 12620, chr14:101489985−101490253−M_values
A431
MCF7
M
8
6
4
2
0
−2
−4
101489950
101490000
101490050
101490100
101490150
101490200
101490250
position (bp)

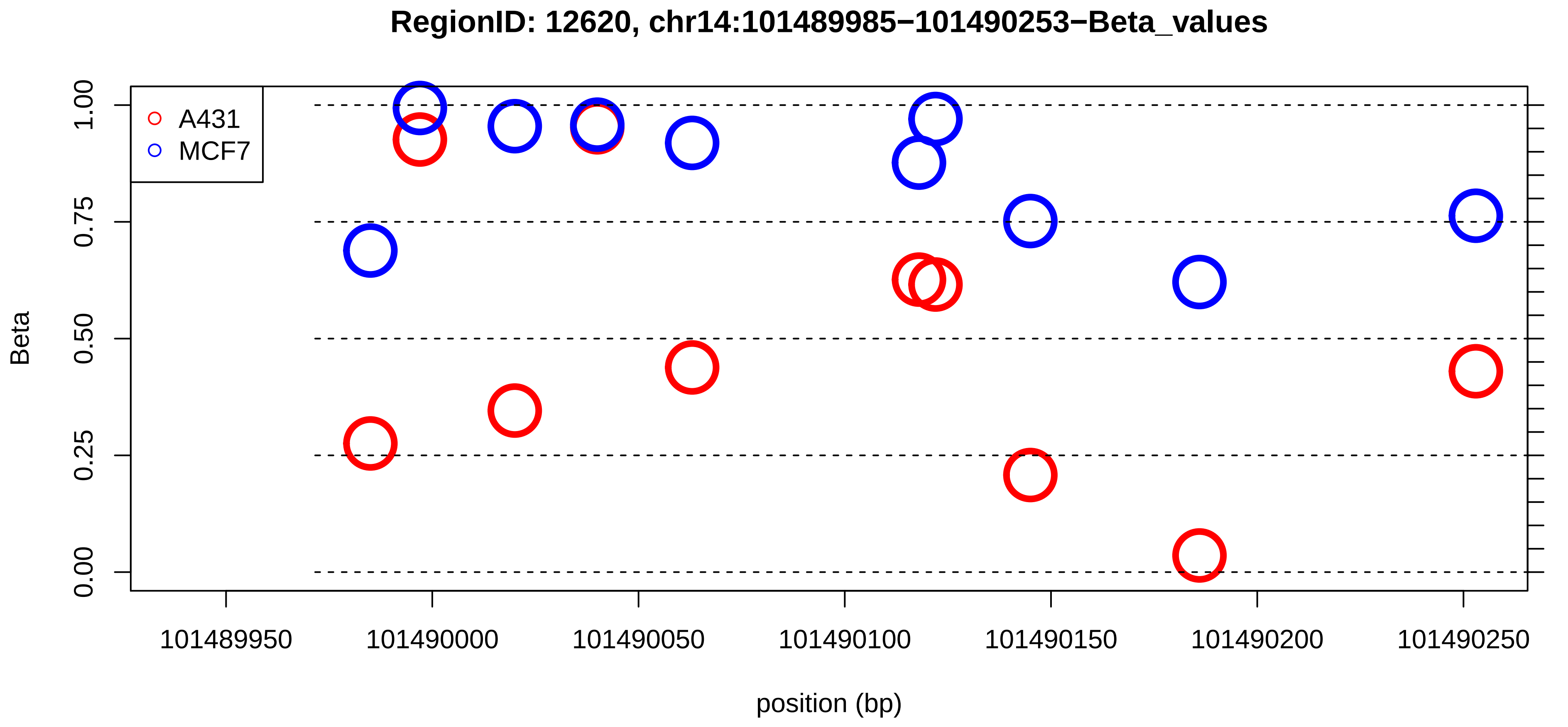
RegionID: 12620, chr14:101489985−101490253−Beta_values
A431
MCF7
Beta
1.00
0.75
0.50
0.25
0.00
101489950
101490000
101490050
101490100
101490150
101490200
101490250
position (bp)

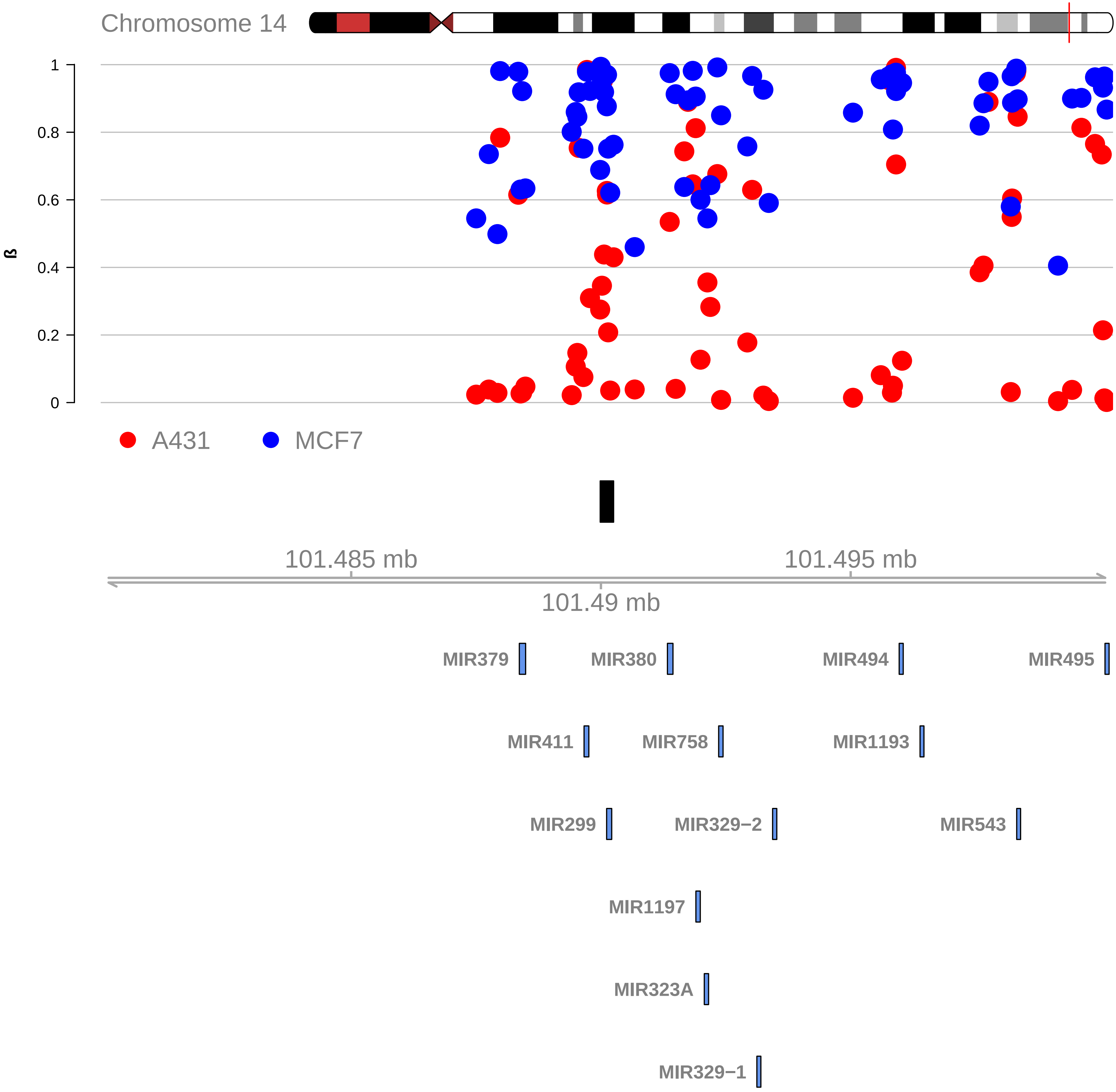

Chromosome 14
1
0.8
0.6
0.4
0.2
0
ß
A431
MCF7
101.485 mb
101.495 mb
101.49 mb
MIR379
MIR380
MIR494
MIR495
MIR411
MIR758
MIR1193
MIR299
MIR329−2
MIR543
MIR1197
MIR323A
MIR329−1
